# Distinct Thalamo-Cortical Controls for Shoulder, Elbow, and Wrist during Locomotion

**DOI:** 10.3389/fncom.2013.00062

**Published:** 2013-05-21

**Authors:** Irina N. Beloozerova, Erik E. Stout, Mikhail G. Sirota

**Affiliations:** ^1^Division of Neurobiology, Barrow Neurological Institute, St. Joseph’s Hospital and Medical CenterPhoenix, AZ, USA

**Keywords:** cat, motor cortex, thalamus, PTN, ventro-lateral thalamus, reticular nucleus of thalamus, accuracy, walking

## Abstract

Recent data from this laboratory on differential controls for the shoulder, elbow, and wrist exerted by the thalamo-cortical network during locomotion is presented, based on experiments involving chronically instrumented cats walking on a flat surface and along a horizontal ladder. The activity of the following three groups of neurons is characterized: (1) neurons of the motor cortex that project to the pyramidal tract (PTNs), (2) neurons of the ventrolateral thalamus (VL), many identified as projecting to the motor cortex (thalamo-cortical neurons, TCs), and (3) neurons of the reticular nucleus of thalamus (RE), which inhibit TCs. Neurons were grouped according to their receptive field into shoulder-, elbow-, and wrist/paw-related categories. During simple locomotion, shoulder-related PTNs were most active in the late stance and early swing, and on the ladder, often increased activity and stride-related modulation while reducing discharge duration. Elbow-related PTNs were most active during late swing/early stance and typically remained similar on the ladder. Wrist-related PTNs were most active during swing, and on the ladder often decreased activity and increased modulation while reducing discharge duration. In the VL, shoulder-related neurons were more active during the transition from swing-to-stance. Elbow-related cells tended to be more active during the transition from stance-to-swing and on the ladder often decreased their activity and increased modulation. Wrist-related neurons were more active throughout the stance phase. In the RE, shoulder-related cells had low discharge rates and depths of modulation and long periods of activity distributed evenly across the cycle. In sharp contrast, wrist/paw-related cells discharged synchronously during the end of stance and swing with short periods of high activity, high modulation, and frequent sleep-type bursting. We conclude that thalamo-cortical network processes information related to different segments of the forelimb differently and exerts distinct controls over the shoulder, elbow, and wrist during locomotion.

## Introduction

Locomotion is one of the most essential and frequently used behaviors. The neural mechanisms that determine the timing and pattern of muscle activity and the coordination of limb movements during locomotion resides in the spinal cord (Shik and Orlovsky, 1976; Grillner and Zangger, 1979; Forssberg et al., 1980a,b). The spinal mechanisms can produce locomotor movements with different rhythms and intensities to adapt to different speeds, different inclines of the support surface, etc. The real environment, however, consists of irregular terrain full of obstacles. Navigating such environments requires land-living animals to control the transfer and placement of their feet accurately. The spinal mechanisms, however, lack information about objects in the outside world that are at a distance. The motor centers of the brain adapt locomotion to the peculiarities of the environment. The motor thalamo-cortical network plays a central role in this adaptation.

In this review we present our recent findings of differential activities of the shoulder, elbow, and wrist-related populations of neurons in the thalamo-cortical network during simple locomotion on flat surface and accurate target stepping along a complex terrain.

Results of a number of biomechanics studies suggest that different segments of the limb are controlled differently. Indeed, limb segments differ in mechanical characteristics, such as dimensions and weight, and differ in their role during movements. Whereas displacements of proximal segments greatly affect the kinematics and kinetics of more distal segments, the influence of a distal segment movement on the mechanical characteristics of proximal segments is much smaller. When Galloway and Koshland (2002) studied point-to-point whole arm movements in humans, they found that movement dynamics differed greatly between the joints. A number of other studies have reported similar data (reviewed in Dounskaia, 2005, 2010). For locomotion, it was shown that the hip angle is an important factor in determining the initiation of the stance-swing phase transition, while angles of distal joints have no effect (Grillner and Rossignol, 1978). In a recent study we found that when stepping has to be accurate during walking along a horizontal ladder, movements in different joints adapt differently to the accuracy demands (Beloozerova et al., 2010). Based on biomechanical evidence, a “leading joint hypothesis” has been advanced proposing that the joints of a limb play roles in movement production according to their mechanical subordination in the joint linkage (Dounskaia, 2005).

Several lines of evidence suggest that the neuronal mechanisms underlying the differences in controls for different forelimb segments are also different. For example, it is well-known that lesions to the pyramidal tract in primates evoke devastating effects on the fine movements of the fingers and wrist, while the disturbances to movements in the proximal joints are much less severe (e.g., Lawrence and Kuypers, 1968). For a reach and prehension task, it was shown that postspike effects of motor cortex pyramidal tract projecting neurons (PTNs) are both more numerous and more prominent on distal muscles as compared to proximal ones (McKiernan et al., 1998). Furthermore, in a study of postnatal development of the forelimb representation in the motor cortex in the cat, Chakrabarty and Martin (2000) have found that the motor map develops in a proximal-to-distal sequence, with shoulder and elbow controls developing earlier than wrist and digit controls. Developmental differences in the controls for different forelimb joints have been reported in humans as well (e.g., Konczak and Dichgans, 1997). Differences were reported also at the single neuron level. While it has been found that nearly all neurons in the shoulder/elbow area of the motor cortex modulate their activity during reaching in accordance with the posture of the arm (Scott and Kalaska, 1997), the activity of only a fraction of neurons in the hand area is wrist posture-related (Kakei et al., 2003). However, the neuronal mechanisms underlying differences in controls for different limb segments have never been explicitly studied until recently. Here we present our data on the differential controls for the shoulder, elbow, and wrist that are used by populations of neurons in the thalamo-cortical network.

**Figure 1:**
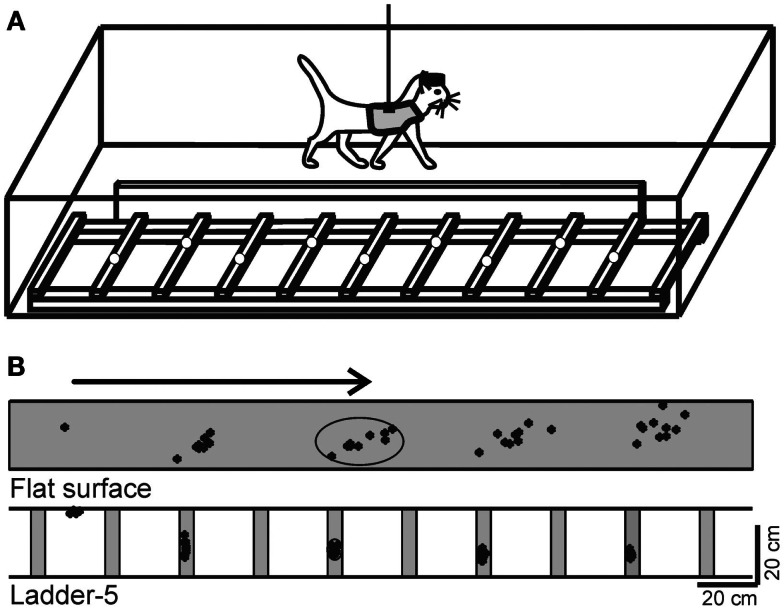
**Locomotion tasks**. **(A)** Cats walked in an experimental box that was divided into two corridors. In one of the corridors, the floor was flat, while the other corridor contained a horizontal ladder. White circles on the crosspieces of the ladder schematically show placements of cat forelimb paws. This schematic drawing is not to scale. **(B)** A typical distribution of right forelimb paw prints recorded from one cat during 10 walking passages though each corridor: on a flat surface (simple locomotion) and along the ladder with crosspieces 5 cm wide (complex locomotion). View from above. The direction of the cat’s progression is shown by the arrow on the top. For simple locomotion, paw prints are adjusted to start in the same position. During the ladder task, the first paw placement during ladder locomotion was between the crosspieces. Ellipses enclose approximate areas in which 95% of paw prints were found. (Adapted with modifications from Beloozerova et al., 2010).

All our experiments were conducted in chronically instrumented cats walking on a flat surface and along a horizontal ladder (Figure 1). Neurons in the motor cortex (MC), all of which were identified as PTNs; neurons in the motor thalamus, most of which were identified as thalamo-cortical projection neurons (TCs) of the ventrolateral nucleus of the thalamus (VL); and inhibitory interneurons of the motor compartment of the reticular nucleus of the thalamus (RE) were recorded (Figure 3). Neurons recorded within each of the MC, VL, and RE were grouped according to the location of their receptive field into shoulder-, elbow, and wrist/paw-related subpopulations. The discharges of these subpopulations within each of the motor centers were compared across the step cycle of simple and ladder locomotion and between the centers. Significant differences were found both between the neuronal groups within each of the motor centers as well as between the centers.

Original data on biomechanics of ladder locomotion were published in Beloozerova et al. (2010); on the activity of the MC – in Stout and Beloozerova (2012); on the activity of the VL – in Marlinski et al. (2012a); and on the activity of the RE – in Marlinski et al. (2012b). Data on biomechanics and the activity of the MC, VL, and RE were all obtained in identical experiments although conducted on different sets of cats. Methods of data collection and spike trains analysis have been described earlier (Beloozerova and Sirota, 1993a; Prilutsky et al., 2005; Beloozerova et al., 2010; Marlinski et al., 2012a,b; Stout and Beloozerova, 2012) and will be briefly outlined below when necessary. All experiments were conducted in accordance with NIH guidelines and with the approval of the Barrow Neurological Institute Animal Care and Use Committee.

## Locomotion Tasks

Two locomotion tasks were used: (1) simple locomotion on a flat surface, and (2) accurate stepping on the crosspieces of a horizontal ladder (Figure 1A). A box 2.5 m long and 0.6 m wide served as an experimental chamber. It had two corridors. In one of the corridors, the floor was flat, while the other corridor contained a horizontal ladder. The crosspieces of the horizontal ladder were flat and 5 cm wide, so that cats had full paw support on the crosspieces. Crosspieces were spaced 25 cm apart, that is, at half of the mean stride length observed in the chamber during locomotion on flat floor (Beloozerova and Sirota, 1993a; Beloozerova et al., 2010). Cats were continuously walking around the chamber, sequentially passing through both corridors, briefly stopping after each round in one of the corners for a food reward.

In our studies we have used a comparison between “non-accurate” locomotion on the flat surface and “accurate” stepping on crosspieces of a horizontal ladder as a tool to reveal the portion of neuronal activity that represents control signals for accurate foot placement during locomotion. It has been demonstrated in several studies that simple locomotion does not require vision and can be successfully performed after the MC has been ablated or inactivated, while locomotion that requires accurate foot placement on complex surfaces, including on a horizontal ladder, depends on vision (Sherk and Fowler, 2001; Beloozerova and Sirota, 2003; Marigold and Patla, 2008), and on the activity of the MC and VL (Trendelenburg, 1911; Liddell and Phillips, 1944; Chambers and Liu, 1957; Beloozerova and Sirota, 1993a, 1998; Metz and Whishaw, 2002; Friel et al., 2007).

**Figure 2:**
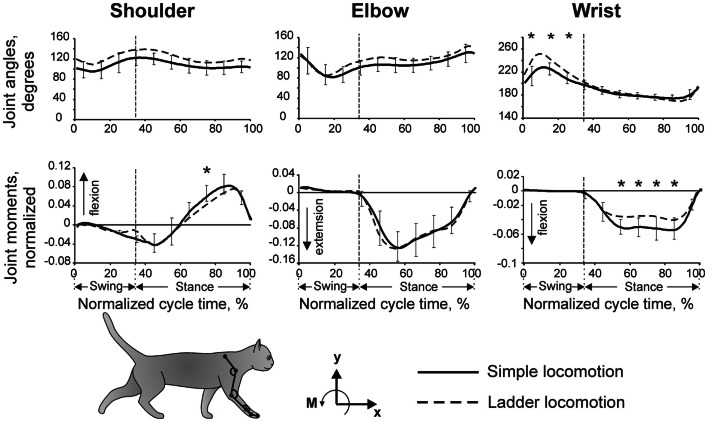
**Forelimb joint angles and moments during simple and ladder locomotion**. Parameters were averaged across five cats. Vertical dashed lines separate the swing and stance phases of the stride. Standard deviations were similar across the two tasks and for clarity are shown only for simple locomotion. Symbol * indicates significant (*p* < 0.05, *post hoc* *t*-test) difference. The cat forelimb model is shown at the bottom. Orientation of each segment was determined as the angle between the negative direction of the vertical axis and the longitudinal segment axis directed from the distal end of the segment to the proximal one. (Adapted with modifications from Beloozerova et al., 2010).

Our detailed examination of biomechanics (229 full-body biomechanical variables were tested) have shown only limited differences between the tasks, apart from paw placement. The variability of paw placement is dramatically smaller during ladder locomotion where, in the direction of progression, it is 5 mm, than during simple unconstrained walking, where it is 70 mm (Figure 1B; Beloozerova et al., 2010). In addition, on the ladder, angles at the distal metacarpophalangeal and metatarsophalangeal joints are slightly different, the wrist is more plantarflexed during swing and its plantar flexion moment during most of stance is lower than during simple locomotion (Figure 2). In contrast to distal joints, there is no significant difference in the values of the proximal joint angles or moments between simple and ladder locomotion (Figure 2). On the ladder cats tilt their neck and head more toward the ground, and the vertical position of the general center of mass and the centers of mass of the neck/head and trunk segments are lower by ∼1–2 cm during ladder as compared to simple locomotion. Out of 229 variables tested, however, there is little else different between simple and ladder locomotion. In particular, the horizontal and vertical displacements of limb segments do not differ significantly between the tasks during most of the step cycle, and the time histories of paw horizontal velocity are symmetric and smooth; there is no statistical difference in the paw velocities between simple and ladder locomotion.

## The Thalamo-Cortical Network for Locomotion

**Figure 3:**
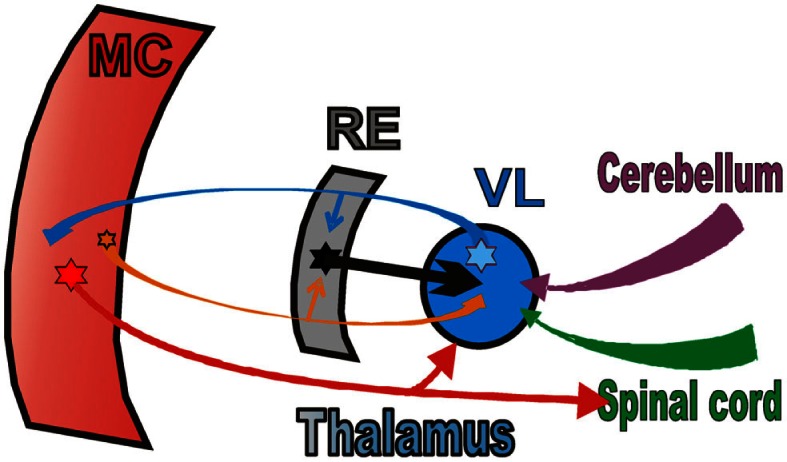
**The scheme of the thalamo-cortical network for locomotion**. MC, motor cortex; RE, motor compartment of the reticular nucleus of thalamus; VL, ventrolateral nucleus of thalamus. Colored stars and arrows show neurons giving excitatory connections. Black star and arrow shows inhibitory neurons and connection.

In this review we will summarize the activities of the three chief elements of the thalamo-cortical network for locomotion (Figure 3). We will first compare and contrast the activities of shoulder-, elbow, and wrist/paw-related neurons of the motor cortex (MC, red plate). All of these neurons were identified as PTNs (red arrow). We will then describe the activity of shoulder-, elbow, and wrist/paw-related neurons of the ventrolateral nucleus of thalamus, a part of the “motor thalamus” (VL, blue circle). The VL receives its major input from the interposed and lateral nuclei of cerebellum (purple arrow), and also receives input from the spinal cord (green arrow). The VL forms the main subcortical input to the MC. Most neurons whose activities are summarized here were identified as thalamo-cortical projection neurons (TCs, blue arrow). TCs synapse on both PTN and interneurons of the MC (Jones, 2007). Finally, we will consider shoulder-, elbow, and wrist/paw-related neurons of the motor compartment of the reticular nucleus of thalamus (RE, gray plate). The RE is a collection of inhibitory neurons that receive inputs from TCs as well as the cortico-thalamic neurons (CT) of the motor cortical layer VI (orange arrow). The RE projects back to the VL, inhibiting it. The RE neurons whose activities are described here received inputs from both the MC and VL. We will not discuss the activity of the CTs of cortical layer VI because, in the MC, they lack somatosensory receptive fields (Sirota et al., 2005), and thus cannot be grouped into shoulder-, elbow, and wrist/paw-related categories.

In our studies, a “relation” of a neuron to control of the shoulder, elbow, or wrist/paw was inferred solely based on receptive field of the neuron. For PTNs, evidence exists that there is a substantial correspondence between a part of the limb, from which a PTN receives somatosensory information, and whose spinal networks it influences (Asanuma et al., 1968; Sakata and Miyamoto, 1968; Rosen and Asanuma, 1972; Murphy et al., 1975). In particular, it was shown that micro-stimulation in the forelimb region of the MC typically produces contraction in single muscles or in small groups of muscles in the area that composes the receptive field at the stimulation site (Asanuma et al., 1968; Sakata and Miyamoto, 1968; Rosen and Asanuma, 1972; Murphy et al., 1975; Armstrong and Drew, 1985a) and affects monosynaptic reflexes of only one or two muscles (Asanuma and Sakata, 1967). Even when series of pulses of 20 μA were used in locomoting subjects, micro-stimulation of a quarter of sites within forelimb motor cortex still affected only one or two muscles (Armstrong and Drew, 1985b). Experiments that used spike-triggered averaging of EMGs in primates showed that although many PTNs excite several motoneuron pools, including those related to muscles on two different segments of the limb or occasionally even across the entire forelimb, approximately half of PTNs influence motoneuron pools that only innervate muscles on one segment of the limb (Buys et al., 1986; McKiernan et al., 1998). For VL and RE neurons no analogous data exist primarily because they are quite remote from muscles. However, the grouping into shoulder-elbow, and wrist/paw-related categories was applied similarly through all elements of the thalamo-cortical network for locomotion. We acknowledge that, at present, it is unknown exactly how cells with different receptive fields in the VL, MC, and RE are connected with each other.

Somatosensory receptive field testing and classification was performed as follows. The receptive fields of neurons were examined in the animals sitting on a comfort pad with their head restrained. Stimulation was produced by palpation of muscle bellies, tendons, and by passive movements of joints. In this review, only neurons with the following somatosensory receptive fields are discussed. (1) The shoulder-related group included neurons responsive only to passive movements in the shoulder joint, and/or palpation of upper back, chest, or lower neck muscles. (2) The elbow-related group included neurons responsive only to passive movements in the elbow joint and/or palpation of upper arm muscles. (3) The wrist-related group included neurons responsive only to passive movements in the wrist joint, and/or palpation of distal arm muscles, and/or to stimulation of the palm or back of the paw. Neurons responsive to movements of toes or claws, those that had receptive field spanning more than one forelimb segment, and neurons without receptive fields were not included.

## Characteristics of Neurons Included in This Review

### PTNs of the MC

**Figure 4:**
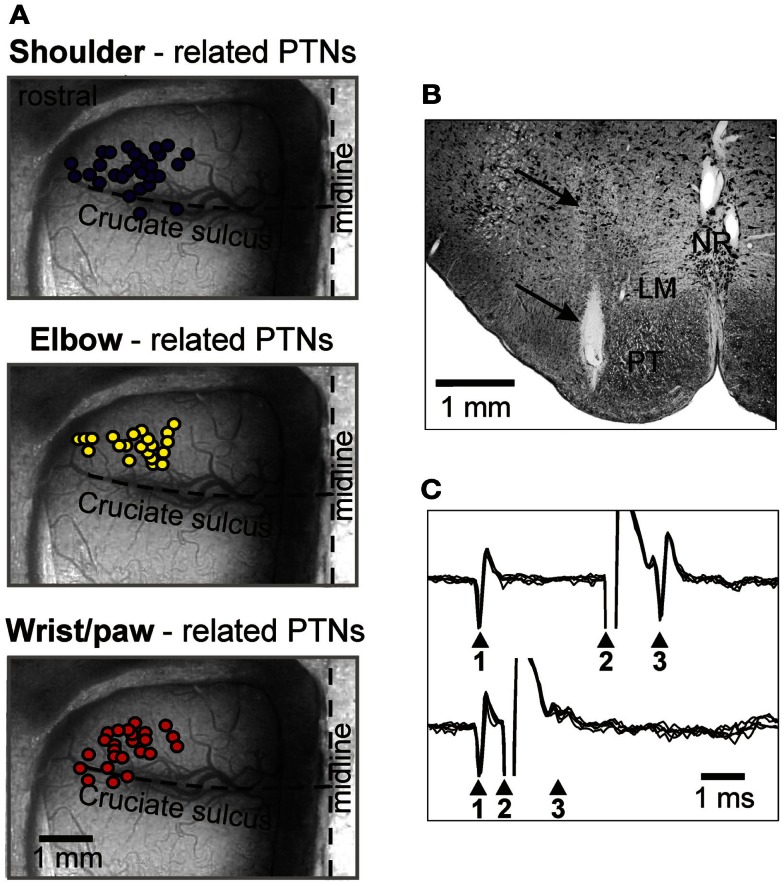
**Location of MC neurons and identification of PTNs**. **(A)** Area of recording in the forelimb representation of the left motor cortex. Microelectrode entry points into the cortex are combined from eight cats and shown by circles on the photograph of the cortex of one cat. Tracks where PTNs with shoulder-related, elbow-related, and wrist-related receptive fields were recorded are shown by purple, yellow, and red circles, respectively. **(B)** Reference electrolytic lesion in the left pyramidal tract. Gliosis surrounding the electrode track and the reference lesion mark are indicated by arrows. Abbreviations: LM, lemniscus medialis; NR, nucleus raphes; PT, pyramidal tract. Frontal 50 μm thick section, cresyl violet stain. **(C)** A collision test determines whether a PTN response was antidromic. Top trace, the PTN spontaneously discharges (arrowhead 1), and the pyramidal tract is stimulated 3 ms later (arrowhead 2). The PTN responds with latency of 1 ms (arrowhead 3). Bottom trace, the PTN spontaneously discharges (arrowhead 1) and the pyramidal tract is stimulated 0.7 ms later (arrowhead 2). PTN does not respond (arrowhead 3) because in 0.7 ms its spontaneous spike was still en route to the site of stimulation in the pyramidal tract, and thus collision/nullification of spontaneous and evoked spikes occurred. (Adapted with modifications from Stout and Beloozerova, 2012).

The activity of 115 PTNs was recorded in eight cats. The vast majority of neurons were sampled from the region of the MC rostral to the cruciate sulcus. In Figure 4A, circles overlaying the cortex schematically show microelectrode entry points into the cortex for tracks in which PTNs with different receptive fields were recorded during locomotion. Receptive fields of all these PTNs were located on the contralateral forelimb and were excitatory. Forty-five PTNs were shoulder-related, 30 were elbow-related, and 40 PTNs were wrist-related. There was extensive spatial overlap between PTN groups.

In their somatosensory responses, most PTNs had some directional preference. Among shoulder-related PTNs, 33% were preferentially responsive to flexion, while 20% were preferentially responsive to extension. The other 43% were responsive to abduction or adduction of the joint, or to palpation of the muscles on the back or chest. Among elbow-receptive PTNs, 37% were preferentially receptive to flexion, and 60% were preferentially receptive to extension. Finally, among wrist-receptive PTNs, 42.5% were receptive to plantar (ventral) flexion of the wrist, while 32.5% were receptive to its dorsal flexion. The remaining 25% of the wrist-related PTNs were receptive to palpation of muscles on the forearm or paw.

To determine whether a MC neuron was projecting through the pyramidal tract, the test for collision of spikes was applied (Bishop et al., 1962; Fuller and Schlag, 1976). It is illustrated in Figures 4B,C. The latencies of antidromic responses of different PTNs to pyramidal tract stimulation varied in the range of 0.4–5.0 ms. Estimated conduction velocities were between 5 and 80 m/s. In shoulder-, elbow-, wrist-related, and non-responsive PTN groups, the proportions of fast and slow conducting neurons were similar.

### VL neurons, including TCs

**Figure 5:**
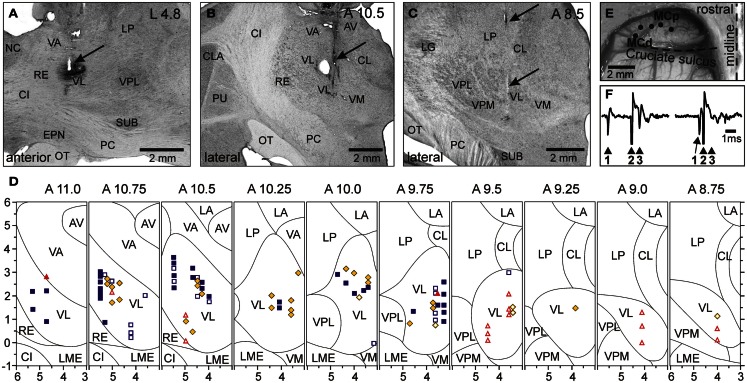
**Location of VL neurons and identification of TCs**. **(A)** The recording site in cat A is shown on a photomicrograph of a parasagittal section of the thalamus. It was located in the rostral VL. The arrow points to the electrolytic lesion mark and the darkened area of tissue filled with WGA-HRP. The site is ∼2 mm caudally to the Nucleus caudatus (NC) of the basal ganglia. **(B)** The recording site in cat B is shown on a photomicrograph of a coronal section of the thalamus. It was positioned in the middle of the VL. The arrow points to the electrolytic lesion mark and darkened area where fluorescent beads were deposited. The caudal part of putamen (PU), a landmark for the anterior-posterior position of the section, is seen laterally. **(C)** The recording site in cat C is shown on a photomicrograph of a coronal section of the thalamus. It was positioned in the caudal VL. The arrows point to a track from a reference electrode. The most rostral aspect of the lateral geniculate body (LG), a landmark for the anterior-posterior position of the section, is visible laterally. **(A–C)** 50 μm thick sections, cresyl violet stain. **(D)** A photograph of the dorsal surface of the left frontal cortex of cat B. Entrance points of stimulation electrodes into the precruciate sulcus are schematically shown by black dots. Electrodes were placed in the paw (the motor cortex distal forelimb representation, MCd), the elbow and shoulder representations (the motor cortex proximal forelimb representation, MCp) as determined by multiunit recording and micro-stimulation procedures. Cru, cruciate sulcus; Pcd, post-cruciate dimple; mAns, medial ansate sulcus. **(E)** A collision test determined whether a neuron response was antidromic. Stimulation of the MC evoked a spike in the neuron with a latency of 0.8 ms. To determine whether this spike was elicited antidromically, on a next trial a spontaneous spike of the neuron was used to trigger MC stimulation with 0.4 ms delay. Stimulation delivered with a delay smaller than the time needed for a spontaneous spike to reach the site of stimulation (that is approximately equal to the latent time of an antidromic spike) was not followed by a response. This indicated a collision of ortho- and antidromically conducted spikes and confirmed the antidromic nature of the evoked spike. **(F)** A reconstruction of positions of individual neurons recorded during locomotion in cats A, B, and C. ■, Purple squares show neurons with somatosensory receptive fields on the shoulder: responding to passive movements in the shoulder joint and/or palpation of muscles on the back or neck; ♦, Yellow diamonds show cells that were activated by movements in the elbow; ▲, Red triangles represent neurons with receptive fields on the wrist or paw. Filled symbols represent neurons with axonal projections to the MC (thalamo-cortical neurons, TCs); open symbols represent neurons whose projections were not identified. Abbreviations: AV, nucleus anterio-ventralis thalami; CI, capsula interna; CL, nucleus centralis lateralis; CLA, claustrum; EPN, nucleus entopeduncularis; LA, nucleus lateralis anterior; LG, lateral geniculate nucleus; LME, lamina medullaris externa thalami; LP, nucleus lateralis posterior; NC, nucleus caudatus; OT, optic tract; PC, pedunculus cerebri; PU, putamen; RE, nucleus reticularis thalami; SUB, nucleus subthalamicus; VA, nucleus ventralis anterior; VL, nucleus ventralis lateralis; VM, nucleus medialis; VPL, nucleus ventralis postero-lateralis; VPM, nucleus ventralis postero-medialis (Adapted with modifications from Marlinski et al., 2012a).

The activity of 97 VL neurons, including 53 TCs, was recorded in three cats. Neurons were sampled starting at the most rostral aspect of the VL that borders the ventral anterior nucleus of the thalamus (VA) at the level of the caudal putamen (Figures 5A,B) and were found caudally up to the level of the rostral aspect of the lateral geniculate body (Figure 5C). In two of cats, retrograde tracers were injected in the area of recoding to determine afferent connections of the areas (WGA-HRP in cat 1, or red fluorescent beads in cat 2). In both cats, numerous labeled neurons were found in the lateral and interposed cerebellar nuclei on the contralateral side, and in cat 1, where recording included the VL-VA border zone, labeled neurons were also found in the lateral half of the ipsilateral entopeduncular nucleus. The receptive fields of all recorded VL neurons were on the contralateral forelimb and were excitatory. Fifty-one cells, including 34 TCs, responded to passive movements of the shoulder joint and/or palpation of muscles on the back or neck. Slightly more than half of these cells showed a directional preference to shoulder movement, and responded better either to flexion or to extension and/or abduction of the joint. Thirty neurons, including 17 TCs, responded to movements in the elbow joint. Almost all of these neurons had a directional preference: half of them responded to flexion and another half to extension of elbow. Sixteen cells, including two TCs, had receptive fields on the paw or wrist. Typically, these neurons responded to pressure on the paw or to the wrist ventral flexion. In Figure 5D, shapes of different colors show estimated locations of all recorded neurons. According to the most often used atlases of the cat diencephalon (Reinoso-Suarez, 1961; Snider and Niemer, 1961; Berman and Jones, 1982), our recordings included the entire rostro-caudal and most of the dorso-ventral extents of the VL. In addition, based on an assessment of receptive fields of the neurons we also concluded that we have covered most of the medio-lateral extent of the forelimb representation in the VL. Neurons that responded to stimulation of different parts of the forelimb were distributed randomly in the VL: there were no clear clusters of shoulder-, elbow-, or wrist paw-related cells.

To determine whether a neuron was projecting to the MC, stimulating electrodes were placed in the layer VI of area 4γ of the distal forelimb representation (paw, MCd) and in the proximal forelimb representation (elbow, shoulder; MCp; Figure 5E), and the test for collision of spikes was applied (Figure 5F; Bishop et al., 1962; Fuller and Schlag, 1976). Thalamo-cortical projection cells (TCs) were distributed fairly evenly throughout the area of recording (Figure 5D). Most TC neurons responded either to stimulation of MCd or MCp, and only few responded to stimulation of both sites. Interestingly, the vast majority (72%) of neurons projecting to MCd had receptive fields on proximal parts of the forelimb, shoulder, or elbow, and only 9% had receptive fields on the wrist or paw. Neurons projecting to MCp had various receptive fields. Latencies of antidromic responses of different TCs varied in the range of 0.5–5.5 ms. Estimated conduction velocities ranged from 5 to 70 m/s.

### RE neurons

**Figure 6:**
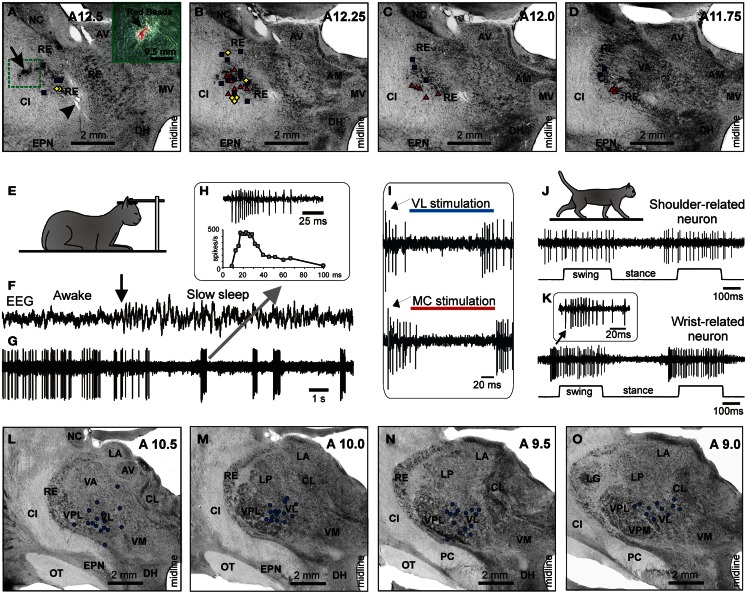
**Location and identification of RE neurons**. **(A–D)** Location of RE neurons recorded during locomotion. Estimated locations of neurons are combined from two cats and are shown by various symbols on frontal sections of thalamus of one of them: ■, Purple squares show neurons with somatosensory receptive fields on the shoulder: responding to passive movements in the shoulder joint and/or palpation of muscles on the back or neck; ♦, Yellow diamonds show cells that were activated by movements in the elbow; ▲, Red triangles represent neurons with receptive fields on the wrist or paw. In **(A)**, an arrowhead is pointing to a reference electrolytic lesion and an arrow indicates the site of injection of red fluorescent beads. **(A)** close-up to the injection site is shown in the insert. Abbreviations: AM, nucleus anterio-medialis; AV, nucleus anterio-ventralis thalami; CI, capsula interna; DH, dorsal hypothalamus; EPN, nucleus entopeduncularis; MV, nucleus medio-ventralis; NC, nucleus caudatus; RE, nucleus reticularis thalami; VA, nucleus ventralis anterior. Frontal 50 μm thick sections, cresyl violet stain. **(E–H)** Identification of RE neurons by characteristic profile of their bursts during sleep. **(E)** Cat sleeping with its head restrained. **(F,G)** An example of activity of a RE neuron while cat is awake and asleep. At the beginning of the record desynchronized activity in EEG indicates that the cat was awake, and the neuron was discharging fairly regularly. The arrow points to the beginning of “spindle waves” in EEG, which are a sign of beginning of slow wave sleep. Shortly thereafter very high frequency irregular bursts separated by long periods of inactivity replaced the regular discharge of the neuron. **(H)** Close-up on a burst. The first interspike interval in this burst was longer than the second one, and the second interval was longer that the third. Several following interspike intervals were of an approximately similar duration, while the last ones were progressively longer. The lower trace shows change of discharge frequency within the burst. Such a burst with ramping up and then winding down firing rate identifies this neuron as belonging to the RE. **(I)** Identification of the motor compartment of the RE by responses of neurons to electrical stimulation of the VL (upper trace) and MC (lower trace). In response to either stimulation the cell generates a short latency burst followed by a period of silence and then by another burst. **(J)** Locomotion-related activity of a representative neuron with shoulder-related receptive field. The activity of this neuron is modulated to strides but does not contain any “sleep-type” busts. **(K)** Accelerating-decelerating frequency “sleep-type” bursting during locomotion in a wrist/paw-related neuron. A burst is shown in the insert at a fast time scale. Such bursts often appeared at the beginning of the locomotion-related activation of this neuron. **(L–O)** Thalamic projections to the area of recording in the RE. Neurons in the VL and VL/VPL border zone in one of the cats where red fluorescent beads were injected in the rostro-lateral part of the explored RE area, retrogradely labeled with red fluorescent beads. Neurons are shown on photomicrographs of frontal sections of the left thalamus ipsilateral to the injection site. Each circle represents one labeled neuron. Abbreviations: CL, nucleus centralis lateralis; LA, nucleus lateralis anterior; LG, lateral geniculate nucleus; LP, nucleus lateralis posterior; OT, optic tract; PC, pedunculus cerebri; VL, nucleus ventralis lateralis; VM, nucleus medialis; VPL, nucleus ventralis postero-lateralis; VPM, nucleus ventralis postero-medialis; other abbreviations are as in Figure 5 (Adapted with modifications from Marlinski et al., 2012b).

Forty-six RE neurons with receptive fields on the contralateral forelimb were recorded from two cats. In Figure 6 the recording sites, combined from both cats, are shown on frontal sections of the thalamus. Cells were collected from the rostro-lateral compartment of the RE at approximate coordinates A 11.75–12.5, L 5.5–7.0, and V 1.0–4.0. The RE was identified by neurons’ characteristic bursts of spikes during sleep (Figures 6E–H). Within these bursts the discharge frequency first ramps up and then winds down (Figure 6H). The motor compartment of the RE was identified by orthodromic responses of the neurons to electrical stimulation of the MC and VL. The overwhelming majority of cells responded vigorously to both stimulations (Figure 8I). A single shock applied to the cortex or VL evoked a sequence of several spikes with interspike intervals of 2–6 ms. Latencies to the first spike were in a range of 1–8 ms, similar for both the cortex and VL. This short latency response was followed by a 120–150 ms period of silence, after which another barrage of high frequency discharge occurred.

In one of the cats, red fluorescent beads were injected into the rostro-lateral part of the explored RE area to reveal the areas of thalamus and cortex that projected to these neurons. In Figure 6A, an arrow points to the site of injection, and Figures 6L–O show locations of neurons retrogradely labeled in the VL. Labeled neurons extended rostro-caudally from A11 to A9, medio-laterally from 3.5 to 5.5, and vertically from 0.5 to 3.0; in addition, labeled neurons were found in a part of the somatosensory ventral posterolateral nucleus (VPL) adjacent to the VL.

Receptive fields of all RE neurons were excitatory. Nineteen cells (41%) were activated by passive movements of the shoulder and/or palpation of muscles on the upper back. Nearly all of these cells had directional preference to shoulder movement, and either responded better to flexion or adduction (13/19) or to extension or abduction of the joint (6/19). Eighteen neurons (39%) had receptive fields on the paw or wrist or responded to passive movements of the wrist, typically in only one direction. The number of neurons responding to passive movements of the elbow was relatively small (20%, 9/46); and all responses were to extension rather than flexion. In Figures 6A–D cells with different receptive fields are depicted with different shapes. There was coarse dorso-ventral topography: cells with receptive fields involving the shoulder were located dorsal to neurons whose receptive fields involved the wrist/paw.

## Examples of Locomotion-Related Activity of Neurons Across the Three Main Elements of the Thalamo-Cortical Network for Locomotion

Analysis of spike trains was performed as follows. The onset of swing phase was taken as the beginning of step cycle. The duration of each step cycle was divided into 20 equal bins, and a phase histogram of spike activity of the neuron in the cycle was generated. The coefficient of stride-related frequency modulation, the “depth” of modulation, dM, that characterizes fluctuation in probability of the spike occurrence, was calculated as dM = (*N*max − *N*min)/*N* × 100%, where *N*max and *N*min are the number of spikes in the maximal and the minimal histogram bin, and *N* is the total number of spikes in the histogram. Neurons with dM > 4% were judged to be stride-related based on an analysis of fluctuation in the activity of neurons in the resting animal (Marlinski et al., 2012a). In stride-related neurons, the portion of the cycle in which the activity level exceeded 25% of the difference between the maximal and minimal frequencies in the histogram was defined as a “period of elevated firing,” or PEF. In neurons with a single PEF, the “preferred phase” of discharge was calculated using circular statistics (Batshelet, 1981; Drew and Doucet, 1991; Fischer, 1993; see also Beloozerova et al., 2003a; Sirota et al., 2005).

**Figure 7:**
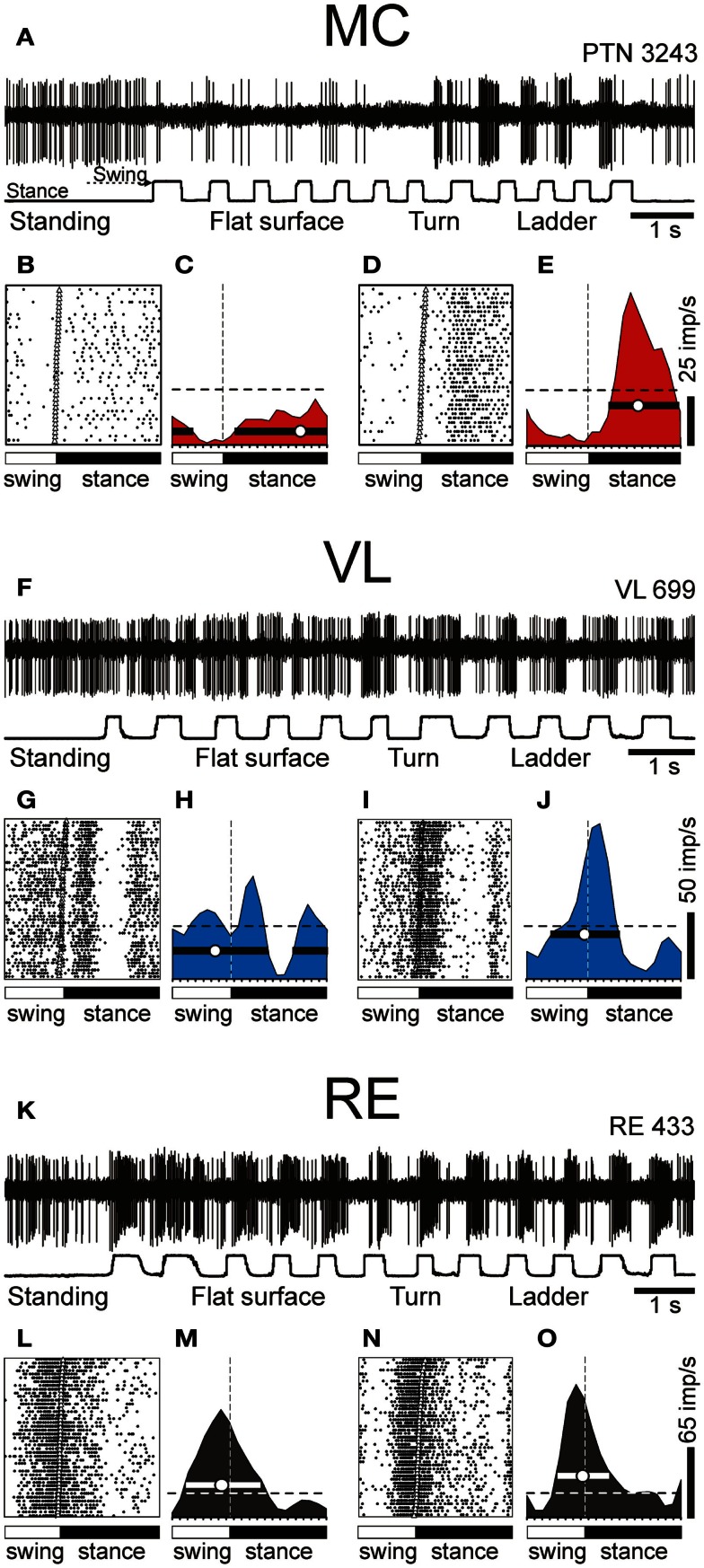
**Example activity of MC, VL, and RE cells during locomotion**. **(A,F,K)** Activity of MC **(A)**, VL **(F)**, and RE **(K)** cells during standing, simple, and ladder locomotion. The bottom trace shows the stance and swing phases of the step cycle of the right forelimb that is contralateral to the recording site in the cortex and thalamus. **(B,C,G,H,L,M)** Activities of the same neurons during simple locomotion are presented as rasters of 37–47 step cycles **(B,G,L)** and as histograms **(C,H,M)**. In the rasters, the duration of step cycles is normalized to 100%, and the rasters are rank-ordered according to the duration of the swing phase. The beginning of the stance phase in each stride is indicated by an open triangle. In the histograms, the horizontal interrupted line shows the level of activity during standing. The horizontal black bar shows the period of elevated firing (PEF) and the circle indicates the preferred phase. **(D,E,I,J,N,O)** Activities of the same neurons during ladder locomotion are presented as rasters **(D,I,N)** and as histograms **(E,J,O)**. (Examples of the activity of MC, VL, and RE neurons are adapted with modifications from Beloozerova et al., 2010; Marlinski et al., 2012a,b, respectively).

An example activity of a PTN during simple and ladder locomotion is shown in Figures 7A–E. At rest, this PTN was activated by passive adduction of the shoulder. The PTN was rather steadily active during standing. When locomotion began, its activity reduced overall but became modulated with respect to the stride: it was greater during stance phase of the stride and smaller during swing. Rasters in Figures 7B,D show that the activity of the PTN was very consistent across strides. The activity is summed in Figures 7C,E showing histograms of PTN firing rate across the step cycle during simple (Figure 7C) and ladder (Figure 7E) locomotion. The PEF is indicated by a black horizontal bar, and the preferred phase is shown by an open circle. Note that during ladder locomotion, the discharge of the neuron during the stance phase was much higher as compared to that during simple locomotion while remaining low during swing phase. Thus, the magnitude of frequency modulation, dM, was larger during ladder locomotion. In addition, the duration of the period of elevated firing, PEF, was shorter by 20% of the cycle.

An example activity of a TC neuron is shown in Figures 7F–J. At rest, this neuron was activated by palpation of muscles around the shoulder. During simple locomotion the neuron discharged throughout all phases of the stride, except for the middle of stance when it was practically silent (Figures 7F–H). This pattern of activity was very consistent across many strides (Figure 7G). The discharge within the PEF varied in intensity, however, forming three small sub-peaks; the maximum discharge rate was 80 spikes/s. During ladder locomotion, rather than discharging throughout most of the stride cycle, the neuron was active almost exclusively around the swing-stance transition (Figures 7F,I,J), but peaked near the same preferred phase as during simple locomotion. Its firing rate reached 118 spikes/s, significantly higher than during simple locomotion (*p* < 0.05, *t*-test), whereas the activity in the trough during stance remained low. Consequently, the magnitude of modulation was larger during ladder than simple locomotion. The duration of the PEF shortened by one half.

An example activity of a RE neuron is shown in Figures 7K–O. At rest, this neuron responded to passive flexion and extension of the shoulder. During locomotion, it was highly active during the end of swing and beginning of stance, and less active at the end of stance phase and beginning of swing. This pattern of activity was consistent across many strides (Figure 7L). The maximum discharge rate of the neuron was 102 spikes/s (Figure 7M). During ladder locomotion, discharge of the neuron during the first half of swing decreased, increasing during the second half of swing to 123 spikes/s. As a result, similarly to both PTN and VL neurons, the magnitude of modulation of the RE neuron’s discharge was larger during ladder than simple locomotion and the PEF was shorter.

We want to note that for none of the MC, VL, or RE, is there a single “typical” neuron with respect to the activity during locomotion. Instead, each of the motor centers contains a variety of neurons that differ in the phases of their discharges during the stride, in the number of PEFs they produce per cycle, in the manner by which they respond to the accuracy demand imposed by the ladder, and other parameters. We did our best to describe these different cell types in our original research reports (Marlinski et al., 2012a,b; Stout and Beloozerova, 2012). In Figure 7 we show neurons with shoulder-related receptive fields that belong to the most populous group of cells: those that discharge a single PEF per cycle and respond to accuracy demand on stepping by increasing the magnitude of their stride-related modulation and by shortening the PEF.

For populations of shoulder-, elbow-, and wrist/paw-related neurons, we will first overview their activities during simple unconstrained locomotion and then consider their discharges during accurate stepping along the horizontal ladder.

## Simple Locomotion: Setting Distinct Frames for the Shoulder, Elbow, and Wrist/Paw Controls

### PTN activity

**Figure 8:**
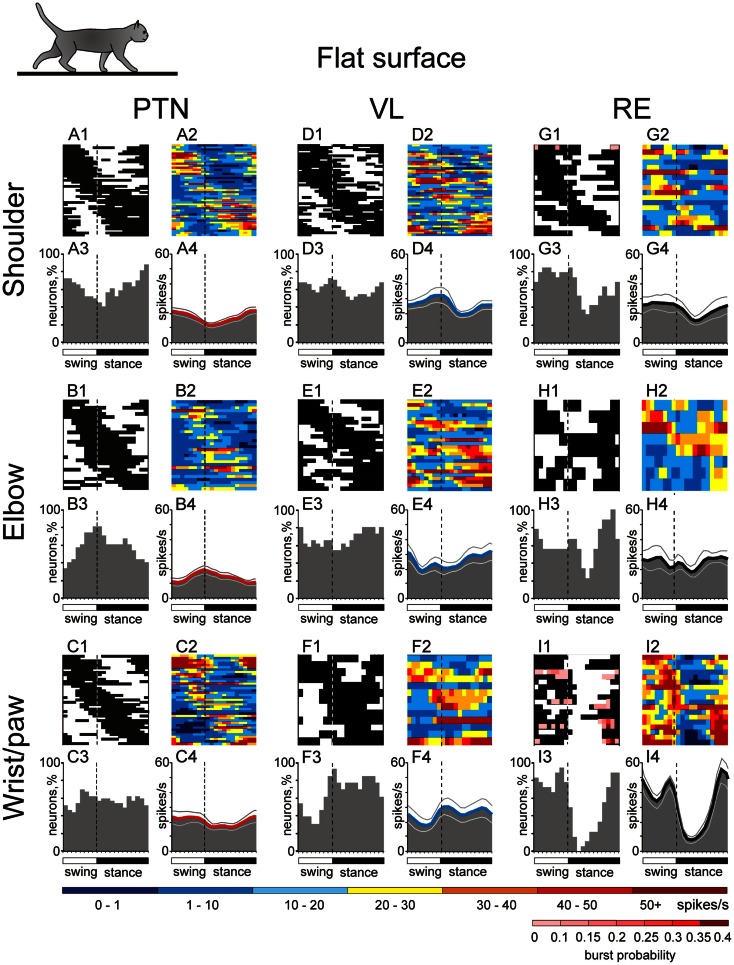
**Activities of the shoulder-, elbow-, and wrist/paw-related cells in the thalamo-cortical network during simple locomotion**. **(A,D,G)** Activity of neurons responsive to movements in the shoulder joint, and/or palpation of back, chest, or neck muscles in the MC **(A)**, VL **(D)**, and RE **(G)**. **(A1,D1,G1)** Phase distribution of PEFs. **(A2,D2,G2)** Corresponding phase distribution of discharge frequencies. The average discharge frequency in each 1/20^th^ portion of the cycle is color-coded according to the scale shown at the bottom. **(A3,D3,G3)** Proportion of active neurons (neurons in their PEFs) in different phases of the step cycle. **(A4,D4,G4)** The mean discharge rate. Thin lines show SEM. Vertical interrupted lines denote end of swing and beginning of stance phase. **(B,E,H)** Activity of neurons responsive to passive movement of the elbow joint in the MC **(B)**, VL **(E)**, and RE **(H)**. **(C,F,I)** Activity of neurons responsive to stimulation of the paw or movement in the wrist joint in the MC **(C)**, VL **(F)**, and RE **(I)**. (Data on the activity of PTNs, VL, and RE neurons are adapted with modifications from Stout and Beloozerova, 2012; Marlinski et al., 2012a,b, respectively).

During simple locomotion, shoulder- and wrist-related PTNs were more active than elbow-related PTNs (18.9 ± 1.3 vs. 13.8 ± 1.7 spikes/s; *t*-test, *p* < 0.05). In 97% of all cells the discharge rate was modulated with respect to the stride: it was greater in one phase of the stride and smaller in another phase. Most PTNs (79%) had one PEF per stride, while 21% had two PEFs. The proportion of two-PEF cells was similar between groups of PTNs with different somatosensory receptive fields, and one- and two-PEF neurons will be considered jointly in this review. The depth of modulation was similar between PTN groups (10.2 ± 0.4%) as was the duration of the PEF (55–60% of the cycle). PEFs of individual PTNs of all groups were distributed across the step cycle. However, this distribution was different between groups (Figure 8, two left columns). Shoulder-related PTNs were more often active during the late stance and early swing (Figures 8A1,3), and their discharge rate was highest during the stance-to-swing transition, at 21.8 ± 2.0 spikes/s (here and below: mean ± SEM), while the firing rate during the opposite phase was 8.4 spikes/s lower (*p* < 0.05, *t*-test; Figures 8A2,4). Elbow-related PTNs were largely active in antiphrase with shoulder-related cells (Figures 8B1,3), discharging during the late swing and early stance at 17.4 ± 2.4 spikes/s, while giving only 10.6 ± 2.1 spikes/s during the opposite phase (Figures 8B2,4). In contrast to both of these groups, PEFs of wrist-related neurons were distributed fairly equally throughout the step cycle (Figures 8C1,3), and their population’ average discharge rate only slightly fluctuated around 20 spikes/s (Figures 8C2,4).

### VL neuron activity

During simple locomotion, the activity of shoulder-, elbow-, and wrist-related VL neurons was similar, and averaged at 23.8 ± 1.4 spikes/s, ∼5 spikes/s higher than the average activity of the most active PTN populations (*t*-test, *p* = 0.01). The activity of 85.5% of neurons, including 87% of TCs, was modulated in the rhythm of strides. Similarly to PTNs, two basic patterns of modulation were seen: one or two PEFs. The one PEF pattern was the most common one (67% of neurons, including 63% TCs). Two PEFs were observed in 31% of cells, including 35% TCs. The proportion of one- and two-PEF cells was similar between groups of VL neurons, and one- and two-PEF cells will be considered jointly below. In the shoulder-related group, the depth of modulation was higher at 9.3 ± 0.6% as compared to either elbow- or wrist/paw-related cells (7.3 ± 0.5%; *p* = 0.02, *t*-test), and the duration of the PEF was shorter (58 ± 3% vs. 65 ± 3% of the cycle; *p* = 0.04, *t*-test). PEFs of individual cells of all groups were distributed across the step cycle. However, as with PTNs, this distribution was different between neuronal groups with different receptive fields (Figure 8, two middle columns).

PEFs of shoulder-related neurons were fairly evenly distributed across the step cycle (Figures 8D1,3); however, neurons with PEFs during end of swing/beginning of stance were more active than other cells (Figure 8D2), and the mean discharge rate of the shoulder-related population was higher during this period, at 27.7 ± 4.0 spikes/s, while the firing rate during mid-stance was 11.2 spikes/s less (*p* = 0.04, *t*-test; Figure 8D4). In contrast, cells of both elbow- and wrist/paw-related groups were most often active during the stance phase (Figures 8E1,3,F1,3). However, while elbow-related neurons attained their maximal population discharge rate at the end of the stance only and during the stance-to-swing transition (Figure 8E4), the mean discharge rate of the wrist/paw-related group was at its highest in the beginning of the stance phase (Figure 8F4). Strikingly, each of VL groups was active largely in anti-phase with their MC counterparts.

### RE neuron activity

During simple locomotion, wrist-related RE neurons were more active then either shoulder- or elbow-related cells (31.4 ± 3.0 vs. 22.6 ± 3.1 spikes/s; *p* < 0.05, *t*-test). The discharge of 96% of all RE neurons was modulated with respect to the stride. Most neurons (74%) had one PEF per step cycle, and 26% had two. Between groups of cells with different somatosensory receptive fields, the proportions of neurons with one and two PEFs were similar. The activity of neurons with receptive fields on the wrist/paw were more modulated than that of either shoulder- or elbow-related groups (12.5 ± 1.1 vs. 8.0 ± 0.6 or 8.4 ± 0.9%; *p* < 0.01, *t*-test), and their PEFs were shorter (54 vs. 66% of the step cycle; *p* = 0.036, *t*-test). As in the PTNs and VL neurons, there was a prominent difference between the phase positions of PEFs of RE cells with different receptive fields. PEFs of wrist/paw-related cells promptly terminated at the end of the swing phase and did not restart before the middle of the stance (Figures 8I1,3). In contrast, PEFs of shoulder- and elbow-related neurons were distributed more evenly across the cycle (Figures 8G1,3,H1,3). Both the wrist/paw- and shoulder-related neurons attained their highest discharge rates during swing and lowest during stance, but the wrist/paw-related population was almost twice as active at the peak as compared to the shoulder-related one (42 and 24 spikes/s, respectively). Overall, RE elbow- and wrist/paw-related neurons were active more or less in anti-phase with their counterparts in the VL, while shoulder-related cells were mostly active in-phase.

In addition to differences in their discharge rates and phase preferences, wrist/paw-, and shoulder-related cells differed sharply in their inclination to produce sleep-type bursts of spikes during locomotion (Figures 6J,K). The activity of a shoulder-related neuron shown in Figure 6J was modulated with respect to the step cycle, but otherwise was rather regular. This firing behavior contrasted sharply with that of a wrist/paw-related neuron shown in Figure 6K. The activity of this neuron was also modulated in relation to the step cycle, however, after a period of silence during stance, it discharged dense bursts of spikes, in which the spike frequency first increased and then decreased. The insert in Figure 6K shows a burst at sufficient temporal resolution to illustrate that its structure during locomotion was similar to the signature RE-type bursts during sleep (Figure 6H). All but one shoulder-related cell had relatively regular firing behavior during locomotion, similar to that of the neuron shown in Figure 6J. In contrast, a significant portion of wrist/paw-related cells (39%, 7/18) discharged sleep-type bursts during walking, similar to those shown in Figure 6K.

### Genesis of locomotion-related activity in the MC, VL, and RE during simple locomotion

We have shown in all three key centers of the thalamo-cortical network for locomotion, MC, VL, and RE, that neurons responsive to stimulation of different forelimb joints are active differently during simple locomotion. While it might be tempting to suggest that these differences are due to differences in the neurons’ somatosensory receptive field characteristics, at least for PTNs, somatosensory information seems not to play a leading role in determining their locomotion-related discharges. Indeed, PTNs with similar receptive fields often discharge during quite different phases of the locomotion cycle (Armstrong and Drew, 1984b). It has been shown that the locomotion-related responses of MC neurons are only slightly affected by changes in the vigor of movements during up- and downslope walking, weight bearing, or alterations in speed (Armstrong and Drew, 1984a; Beloozerova and Sirota, 1993b) – changes that most certainly cause significant changes to proprioceptive afferentation. With regard to cutaneous input, Armstrong and Drew (1984b) have demonstrated that in the MC, neurons with cutaneous receptive fields, including on the forefoot, still rhythmically discharge during locomotion with a similar phasing relative to the step cycle when their response to mechanical stimulation in the receptive field is temporarily reduced or abolished by local anesthesia of the skin. In our recent study we found that the great majority of PTNs with direction-specific receptive fields did not show any particular preference to discharge in-phase with stimulation of their receptive field during locomotion (Stout and Beloozerova, 2012). Similarly poor relationships between phasing of task-related discharges and directional specificity of PTN resting receptive fields were reported in previous studies from our and other laboratories (Armstrong and Drew, 1984b; Drew, 1993; Beloozerova et al., 2003b, 2005; Karayannidou et al., 2008).

For VL and RE neurons, the above experiments have not been conducted; however, one can argue that discharges of neurons in these thalamic nuclei during simple locomotion are likewise, at the very least, not entirely driven by stimulation of somatosensory receptive fields. In our studies we did not find any simple correlation between neuronal responses to somatosensory stimulation in the quiescent animal and preferred phases of VL neurons activity during locomotion (Marlinski et al., 2012a). In decerebrated cats, it was found that the cerebellum plays the pivotal role in driving locomotion-related discharges in the neurons of subcortical motor centers, including neurons of the red nucleus, vestibular nuclei, and the neurons of the reticular formation giving rise to the reticulo-spinal tract (Orlovsky, 1970, 1972a,b; reviewed in Arshavsky et al., 1986; Orlovsky et al., 1999). For these centers, the role of direct afferentation from the spinal cord for periodic modulation of activity during locomotion is minimal, because in the majority of their neurons, locomotion-related modulation disappears after removal of the cerebellum in the decerebrate preparation. It can be expected that the VL, as a subcortical motor nucleus receiving direct connections from the cerebellum, does not differ in this respect from the brainstem motor centers. It is important to stress that the locomotion-related output of the cerebellum during simple locomotion is almost exclusively formed on the basis of information that is obtained from the spinal locomotor CPG (rev. in Arshavsky et al., 1986 and Orlovsky et al., 1999). The VL receives this information. All deep cerebellar nuclei project to the area of VL that we explored (Rinvik and Grofová, 1974; Rispal-Padel and Grangetto, 1977; Angaut, 1979; Nakano et al., 1980; Ilinsky and Kultas-Ilinsky, 1984; Evrard and Craig, 2008; Marlinski et al., 2012a), and it was shown that all these nuclei house neurons whose activity is strongly step-related during locomotion, with characteristics that are very suitable for driving locomotion-related activity in the VL (Orlovsky, 1972c; Armstrong and Edgley, 1984, 1988; Beloozerova and Sirota, 1998; Nilaweera and Beloozerova, 2009).

Signals to the motor compartment of the RE come from collaterals of VL TCs and the collaterals of MC cortico-thalamic (CT) neurons of layer VI (Figure 3; rev. in Jones, 2007). A comparison of locomotion-related discharges in these two regiones (Sirota et al., 2005 for CTs; Marlinski et al., 2012a for TCs) shows that the activity of the RE is very similar to that of the VL and appears to be predominantly driven by it (see Marlinski et al., 2012b for a detailed discussion). Therefore, one can conclude that if during simple locomotion VL neurons are, at least to a significant extent, driven by the spinal locomotor CPG, so too are the neurons of the RE.

If the activity of MC, VL, and RE neurons is influenced by signals from the spinal locomotor CPG, then this influence is quite different for neurons associated with different joints of the forelimb (Figure 8), as we found that these cells tend to discharge differently during simple locomotion. Namely, for VL neurons that are the “entry” elements of the network (Figure 3), the influence from the CPG onto shoulder- and wrist/paw-related groups is maximal during the swing-to-stance transition, and onto the elbow-related group during the opposite phase. For RE neurons, which form a feedback inhibition loop for the VL, the influence from the CPG, although arriving in a similar phase of the stride, greatly differs in magnitude between the wrist/paw-related group and the shoulder- and elbow-related groups. For PTNs, which are the output elements of the network, the influence from the CPG onto the shoulder-related group is maximal during the stance-to-swing-transition, during the opposite phase for the elbow-related group, and roughly even throughout the step cycle for the wrist-related group.

### Function of locomotion-related activity in the MC, VL, and RE during simple locomotion

Many studies have demonstrated that the MC does not exert decisive control over simple locomotion. Analogous data was also reported for the VL (Fabre and Buser, 1979; Beloozerova and Sirota, 1993a). In our earlier publication we have suggested that the stride-related modulation of the activity that MC neurons exhibit during simple locomotion has an informational character, allowing these neurons, if a need arises, to influence the spinal locomotor mechanism for correction of movements without disturbing the overall stepping rhythm (Beloozerova and Sirota, 1993a). We have later extended this hypothesis to both the VL and RE (Marlinski et al., 2012b).

It is important to understand how setting of permissible “windows of influence” takes place. Locomotion-related modulation of PTNs appears to be primarily caused by the activity of the VL, the main subcortical input to the MC. The general importance of this input for MC activity is well-known (Massion, 1976; Fabre-Thorpe and Levesque, 1991; Shinoda et al., 1993; Horne and Butler, 1995; Steriade, 1995; Destexhe and Sejnowski, 2001); however, the contribution of the VL to the transmission of locomotion-related signals has been not researched before. We found that discharges of 92% of VL neurons are modulated in the rhythm of strides with cells expressing one- and two-PEF patterns in proportions that are close to those seen in PTNs (Armstrong and Drew, 1984a; Beloozerova and Sirota, 1993a; Drew, 1993; Stout and Beloozerova, 2012). Thus, TCs can contribute to the activity in the MC during locomotion. However, in the four major characteristics of locomotion-related activity: mean discharge frequency, depth of frequency modulation, duration of activity bursts, and their stride phase distribution, there are two notable differences in the activity of VL neurons as compared to PTNs. The average depth of modulation is lower in the VL: 7.3–9.3 ± 0.5% vs. 10.2 ± 0.4% (*p* < 0.05, *t*-test), and the discharge within the activity bursts is typically more variable (Marlinski et al., 2012a). That is, stride-related responses of VL neurons are less phase-specific as compared to those of PTNs. This agrees with previous findings of a weaker directional specificity of VL neurons discharges during arm and wrist movements as compared to that of neurons in the motor cortex (Strick, 1976; Kurata, 2005), as well as with the well-known fact that, in the visual system, the responses of neurons in the lateral geniculate nucleus are less specific to visual stimuli than those of cells in the visual cortex (e.g., Tsao and Livingstone, 2008). This means that even during simple locomotion, the MC integrates its own information processing into signals received from the VL and likely takes into account other, predominantly cortical, inputs.

In addition to general differences in VL and PTN activities during locomotion, each of the shoulder-, elbow-, and wrist-related VL groups discharges in anti-phase with their respective PTN counterpart much of the time (Figures 8 and 11). This can have several reasons. First, it is possible that TCs direct their main output to PTNs not with a similar, but rather a dissimilar receptive field. Using electrical stimulation of the MC we found that the vast majority (72%) of TCs projecting to distal forelimb representation in the MC had receptive fields on proximal parts of the forelimb. Correspondingly, shoulder-related TC neuron activity is roughly in-phase with that of wrist/paw-related PTNs (Figures 8C4,D4). Although we did not find any other statistically significant crossed projections, elbow-related TCs activity was in-phase with that of shoulder-related PTNs, and wrist-related TCs as a group were active roughly in-phase with elbow-related PTNs.

A second explanation for the generally antiphasic activity of VL and PTNs subpopulations with similar receptive fields is that, in analogy with the somatosensory cortex where TCs powerfully excite inhibitory interneurons (Swadlow, 2002), PTNs may receive their main input from TCs not directly but via an inhibitory cortical network. This is quite plausible because putative inhibitory interneurons with suitable locomotion-related properties have been seen in the MC (Beloozerova et al., 2003a,c; rabbit, cat; Murray and Keller, 2011, rat). GABAergic inhibitory interneurons are thought to be involved in regulating both spatial and temporal response properties of cortical neurons (Sillito, 1975; Hicks and Dykes, 1983; Dykes et al., 1984), and it was demonstrated that they importantly participate in motor-related responses of PTNs as reduction of cortical GABA_A_ inhibition enhances PTN activity during voluntary movements (Matsumura et al., 1992) and postural corrections (Tamarova et al., 2007).

Finally, since among both VL and PTN subpopulations there are neurons that are active in any phase of the stride, it is possible that although gross populational activities of VL and PTNs are in anti-phase, individual TC neurons influence those PTNs with which they are active in-phase. This will imply that VL neurons active during different phases of the stride have different divergence/convergence ratios for different PTNs. For example, in the wrist/paw domain, the few TC neurons active during swing diverge and powerfully drive many PTNs, while the many TCs that are active during stance converge upon similar overall numbers of PTNs, but drive them less powerfully (Figures 8C3,4,**F3**,4). These possibilities of fine organization of TC to PTN projection can and should be tested experimentally.

The activity of the VL is shaped by operation of the inhibitory feedback through the RE (Figure 3). While a wealth of information is available on the properties of RE neurons in brain slices, in anesthetized animals, and during sleep (Steriade et al., 1990; McCormick and Bal, 1997; Funke and Eysel, 1998; McCormick and Contreras, 2001; Hartings et al., 2003; Lam and Sherman, 2005, 2007, 2011; Cotillon-Williams et al., 2008; Sillito and Jones, 2008), the involvement of the RE in the production of movements has not been researched until recently (Marlinski et al., 2012b). In our studies we found that the activity of 90% of RE neurons is step phase-related during locomotion. The fact that the activity of the RE, at both the individual and population level, changes with the phase of the stride indicates that during different stride phases RE neurons exerts different influences upon the VL. The activity of all RE subpopulations is more intense during late stance and swing as compared to early stance (Figures 8 and 11). This means that their target VL neurons are most inhibited during late stance and swing, thus allowing only the strongest ascending signals to pass through and reach the MC during these periods. A blockade of thalamic transmission permits other inputs to the MC to gain a greater contribution to the formation of cortical output during late stance and throughout swing phase. In contrast, during the early stance phase, when the activity of RE neurons is the lowest and thus their target VL cells are disinhibited, more ascending information passes through thalamus to the MC allowing the thalamus to provide a larger contribution to the cortical output during this period.

RE neurons with receptive fields on different segments of the forelimb, likely related to control of different segments of the limb, act differently during locomotion. Wrist/paw-related neurons, which are located ventrally in the nucleus, greatly exceed both shoulder- and elbow-related cells in the magnitude of their population activity modulation (Figures 8 and 11). They also have the highest discharge rates and greatest depths of frequency modulation in discharges of individual neurons, and are prone to high frequency bursting. The shoulder-related cells, which are located dorsally in the nucleus, have the lowest discharge rates and depths of modulation and rarely if ever burst. Thus, the VL-to-MC signal transmission in the distal limb domain is the most heavily influenced by the RE and is least influenced in the proximal limb domain.

## Ladder Locomotion: Exerting Differential Controls Over Shoulder, Elbow, and Wrist/Paw for Achievement of Accurate Stepping

The ladder adds accuracy requirements to the locomotion task. On the ladder, cats are forced to constrain their paw placement to the raised crosspieces. They step accurately on their tops, showing much less spatial variability in feet placement as compared to simple locomotion (Beloozerova et al., 2010; Figure 1B). It has been demonstrated that walking with accurate stepping requires visual control (Sherk and Fowler, 2001; Beloozerova and Sirota, 2003; Marigold and Patla, 2008) and the activity of the MC and VL to be successful (Trendelenburg, 1911; Liddell and Phillips, 1944; Chambers and Liu, 1957; Beloozerova and Sirota, 1988, 1993a, 1998; Metz and Whishaw, 2002; Friel et al., 2007). In our experiments, all neurons that were tested during walking on the flat surface were also tested during locomotion along the ladder.

### PTN activity

Upon transition from simple to ladder locomotion, 97% of PTNs changed at least one characteristic of their activity, and 76% changed two or more. During ladder locomotion, high proportions of PTNs in all somatosensory response groups, 27–42% depending on the group, increased their average discharge rate as compared to simple walking, on average by 99 ± 74%. Overall, fewer cells decreased the activity. Wrist- and elbow-related groups differed sharply, however: wrist-related PTNs had a fair number of cells with diminishing activity (40%), while the elbow-related group had only few (15%). In result, the average discharge rate of elbow-related group increased and became similar to that of shoulder- and wrist/paw-related PTNs. The average rate for all PTNs was 19.3 ± 1.2 spikes/s.

**Figure 9:**
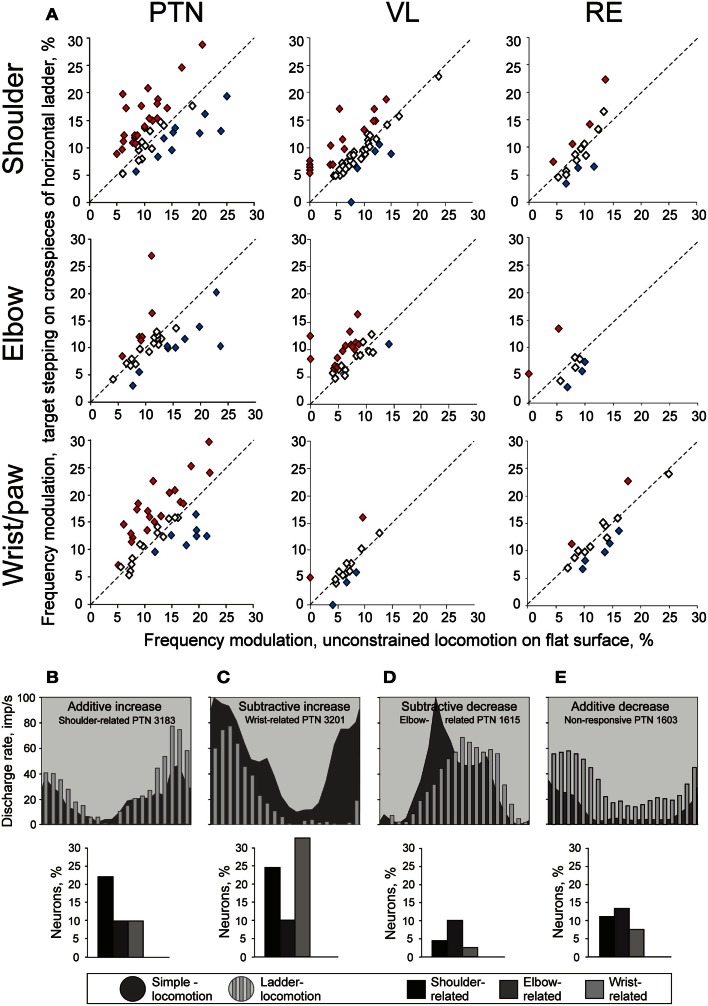
**Change in the depth of frequency modulation upon transition from simple to ladder locomotion**. **(A)** Comparison of depth of modulation in the activity of individual MC, VL, and RE neurons. The abscissa and ordinate of each point show the values of the depth of modulation of a neuron during simple and ladder locomotion, respectively. Neurons whose depths of modulation were statistically significantly different during the two tasks are shown with filled diamonds, the other ones are shown with open diamonds. **(B–E)** Typical changes in the depth of modulation upon transition from simple to ladder locomotion in PTNs. The area histograms show the activity of typical PTNs during simple locomotion, and the bar histograms show activity of the same PTNs during ladder locomotion. Bar graphs beneath the histograms show the proportion of PTNs from each group exhibiting that type of modulation change. **(B):** Increase in the depth of modulation by additive mechanism. **(C)** Increase in the depth of modulation by subtractive mechanism. **(D)** Decrease in the depth of modulation by subtractive mechanism. **(E)** Decrease in the depth of modulation by additive mechanism. (Adapted with modifications from Stout and Beloozerova, 2012).

The activity of all but three PTNs was stride-related during ladder locomotion. The average depth of modulation was 11.4 ± 0.4%. The same two patterns of modulation were observed in proportions similar to those seen during simple locomotion. Half of shoulder- and wrist-related PTNs increased the depth of modulation, on average by 62 ± 44% (Figure 9A). To do this, wrist/paw-related PTNs most commonly decreased discharge rate during the inter-PEF interval, while shoulder-related neurons could either increase it within the PEF or decrease in-between the PEFs (Figures 9B,C). Decreases of modulation also occurred in these neurons, but only half as frequently. In contrast, a typical response of elbow-related PTNs to the ladder task was a decrease of modulation depth (Figure 9A), typically by a decrease in the firing rate during the PEF (Figure 9D). About one third of shoulder- and wrist-related PTNs decreased the duration of their PEF, on average by ∼40%, but typically kept the same number of PEFs. In contrast, the elbow-related neurons typically did not change the PEF’s duration, but tended to change the number of PEFs by either increasing or decreasing it.

**Figure 10:**
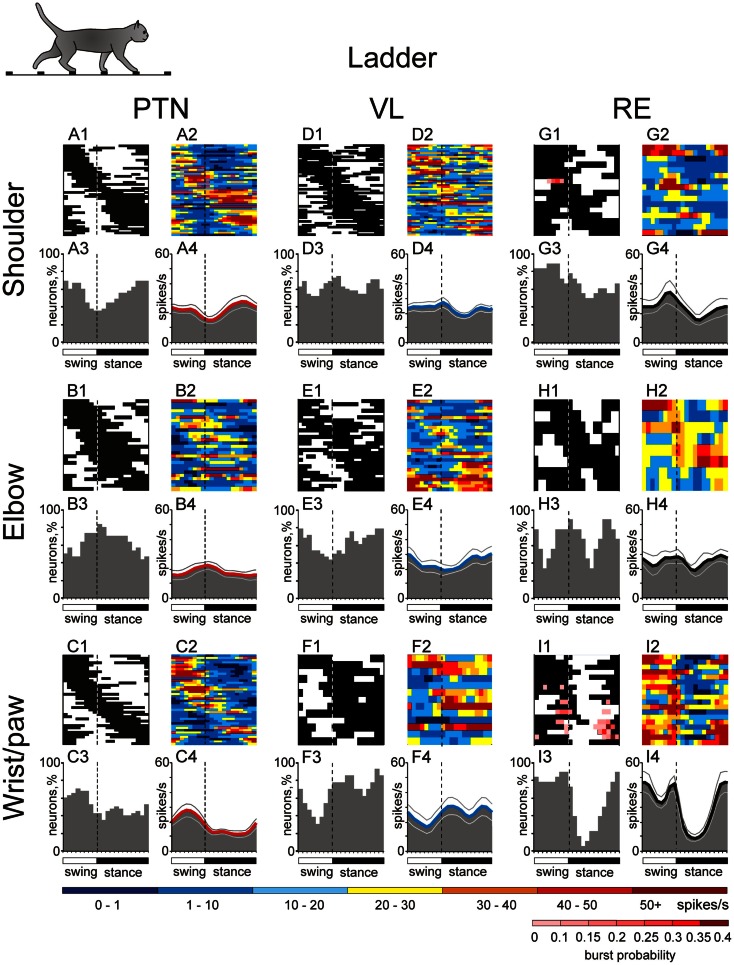
**Activities of the shoulder-, elbow-, and wrist/paw-related cells in the thalamo-cortical network during ladder locomotion**. **(A,D,G)** Activity of neurons responsive to movements in the shoulder joint, and/or palpation of back, chest, or neck muscles in the MC **(A)**, VL **(D)**, and RE **(G)**. **(A1,D1,G1)** Phase distribution of PEFs. (**A2,D2,G2)** Corresponding phase distribution of discharge frequencies. The average discharge frequency in each 1/20^th^ portion of the cycle is color-coded according to the scale shown at the bottom. **(A3,D3,G3)** Proportion of active neurons (neurons in their PEFs) in different phases of the step cycle. **(A4,D4,G4)** The mean discharge rate. Thin lines show SEM. Vertical interrupted lines denote end of swing and beginning of stance phase. **(B,E,H)** Activity of neurons responsive to passive movement of the elbow joint in the MC **(B)**, VL **(E)**, and RE **(H)**. **(C,F,I)** Activity of neurons responsive to stimulation of the paw or movement in the wrist joint in the MC **(C)**, VL **(F)**, and RE **(I)**. (Data on the activity of PTNs, VL, and RE neurons are adapted with modifications from Stout and Beloozerova, 2012; Marlinski et al., 2012a,b, respectively).

A number of PTNs, especially within the elbow-related group, changed their preferred phases of the activity by either discharging earlier or later in the cycle. However, the phasing preferences of the entire shoulder- and elbow-related subpopulations during ladder locomotion remained largely similar to those during simple locomotion (Figures 8 and 10). In shoulder-related PTNs the mean discharge rate during stance-to-swing transition slightly rose to 24.4 ± 2.9 spikes/s; however, the activity during the opposite phase also rose, reaching 16.1 ± 2.4 spikes/s. Elbow-related PTNs still had a tendency to discharge more intensively during the swing-to-stance transition (Figures 8 and 10). In stark contrast to those groups, wrist-related PTNs developed a strong phase preference. While during simple locomotion this group showed only a subtle tendency to discharge more intensively during swing, during ladder locomotion this preference became pronounced (Figures 8 and 10). The discharge during swing was now slightly higher and, in addition, the discharge rate during stance substantially decreased. So, the difference in the discharge rate between swing and stance of wrist-related PTNs was 14.6 spikes/s during ladder locomotion.

### VL neuron activity

Upon transition from simple to ladder locomotion, 79% of VL neurons changed at least one characteristic of their activity. One third of cells changed the discharge rate by either increasing or decreasing it by 51 ± 7% on average. While the average discharge rate of shoulder-, elbow-, and wrist-related neurons remained similar to that during simple locomotion (23–25.5 spikes/s), the elbow-related VL group was different from both other groups in that it had significantly more neurons whose activity diminished upon transition from simple to ladder locomotion (*p* = 0.01, χ^2^ test). This change in the activity of VL elbow-related neurons directly opposed that of elbow-related PTNs.

The activity of 92% of all VL neurons was step-related during ladder locomotion, with eight neurons becoming step cycle-modulated only during this complex task. The average depth of modulation was 9.1 ± 0.4%. The same two patterns of discharge modulation as during simple locomotion were expressed: the one PEF (63% of neurons) and the two-PEF (34% of neurons) patterns. In the shoulder-related group, 32% of cells increased and 10% decreased the depth of modulation, but the average depth of modulation for this subpopulation did not significantly change. In contrast, half of the elbow-related cells increased the depth of modulation, on average by 60 ± 7% (Figure 9A), and in result, the depth of modulation of the elbow-related group increased to 9.5 ± 0.6%. In the wrist-related group, only 15% of cells increased modulation and 15% decreased it, and the average modulation of wrist/paw-related cells remained low. The duration of the PEF was similar across the three VL neuronal groups, averaging 61 ± 1.5% of the cycle, however, in about one third of cells the number of PEFs per cycle changed. Elbow-related neurons differed from both other groups by almost always increasing the number of PEFs on the ladder from one to two, while shoulder- and wrist/paw-related cells more often decreased it from two to one. In approximately one quarter of neurons that were modulated with one PEF during both locomotion tasks, regardless of their receptive field, the preferred phase of the activity on the ladder was different from that during simple locomotion.

Ventrolateral thalamus neurons with receptive fields involving different joints tended to have their PEF in different phases of the step cycle (Figure 10, two middle columns). Despite changes in preferred phases of activity of individual neurons, populations’ activity distributions were generally similar to those seen during simple locomotion. Shoulder-related neurons were more active during the transitions from swing-to-stance phase, and the mean discharge rate of the stride-related population was higher during this period, at 27.0 ± 3.3 spikes/s, while the firing rate during mid-stance was 10 spikes/s less (Figure 10D4). Elbow-related neurons tended to be more active in the opposite phase, reaching maximum in the activity at 30 ± 5.0 spikes/s during the late stance and early swing (Figure 10E4). Wrist-related neurons were more active throughout stance at 25–30 spikes/s while discharging 10–15 spikes/s less during mid swing (Figure 10F4).

### RE neuron activity

Upon transition from simple to ladder locomotion, 75% of RE neurons changed at least one characteristic of their activity (Figure 9A). During ladder locomotion, wrist-related RE neurons still tended to be more active then either shoulder- or elbow-related cells (29 ± 3.4 vs. 24.5 ± 3.0 spikes/s). The discharge of 91% of all RE cells was modulated with respect to the stride, and as with the MC and VL neurons, the same two patterns of modulation were observed in proportions similar to those seen during simple locomotion.

There were substantial differences in the activity between neurons with different receptive fields (Figure 10). As with the VL populations, distributions were generally similar to those seen during simple locomotion. PEFs of shoulder-related cells were distributed rather evenly across the cycle (Figures 10G1–4), and their average discharge rate was relatively low (23 ± 3.3 spikes/s). They also had low average depth of modulation (8 ± 1%) and long PEFs (70 ± 3% of the cycle). In contrast, wrist/paw-related cells discharged most intensively during the swing and end of stance, generally sparing the first half of stance (Figures 10I1–4). They also tended to be more active (29 ± 3.4 spikes/s), were much more modulated (12.4 ± 1.2%), and exhibited shorter PEFs than neurons of any other group (55 ± 4.5% of the cycle). In addition, wrist/paw- and shoulder-related cells still differed dramatically in production of sleep-type spike bursts. The most frequently bursting wrist/paw-related cell generated a burst nearly every third stride, while shoulder- and elbow-related generated very few if any. Three wrist-related neurons had a significantly higher probability to discharge a sleep-like burst during ladder than simple locomotion (*p* = 0.001, *t*-test). The activity characteristics of elbow-related neurons were in-between of those of shoulder- and wrist/paw-related cells (Figures 10H1–4).

### Distinct MC controls for shoulder, elbow, and wrist during complex locomotion

It is clear that the MC plays a critical role in the control of accurate stepping, as precise positioning of limbs is nearly impossible after destruction of the MC or even its short-lasting inactivation (Trendelenburg, 1911; Liddell and Phillips, 1944; Chambers and Liu, 1957; Beloozerova and Sirota, 1988, 1993a; Metz and Whishaw, 2002; Friel et al., 2007). In cats walking on a treadmill belt, it was shown that the activity of many neurons in the MC changes periodically according to the step cycle, and significantly increases during unexpected perturbations and voluntary gait modifications (Armstrong and Drew, 1984a; Drew, 1993; Widajewicz et al., 1995; Drew et al., 1996). In our earlier work, we found that when paw positioning on the surface was restricted such that visually guided adaptation of gait was required to place the paws accurately, the activity of 60–70% of the neurons in the MC, depending on the task, changed dramatically as compared to walking on the flat surface, and the changes in neuronal activity increased as the requirements for accurate foot placement became increasingly demanding (Beloozerova and Sirota, 1993a). Later, we additionally found that, as accuracy demand on stepping progressively increases, many neurons in the MC progressively refine their discharge timing, producing activity more precisely in a specific and restricted phase of the stride (Beloozerova et al., 2010).

Several lines of evidence indicate that the differences in MC activity during simple and ladder locomotion reflect different modes of cortical descending control during these tasks, not a difference in the afferent signals. First, as discussed above, afferent signals appear to play little role in driving locomotion-related responses in MC neurons (Armstrong and Drew, 1984a,b; Beloozerova and Sirota, 1993a,b; Stout and Beloozerova, 2012). Second, in our recent study we have examined 229 full-body biomechanical variables of cats walking on the flat surface and along a horizontal ladder with flat rungs placed at a convenient for the cat distance (Beloozerova et al., 2010). We found that on such ladder, cats step on support surface with much less spatial variability (Figure 1B) but the overwhelming majority of other biomechanical variables do not differ between the tasks. This suggests that afferentation received by the MC during simple and ladder locomotion may be very similar. While it was shown that the level of fusimotor activity is often higher during difficult motor tasks, especially those that are novel, strenuous, or are associated with high degree of uncertainty (Prochazka et al., 1988; Hulliger et al., 1989), our ladder locomotion task was well practiced, entirely predictable, and, judging from levels of EMG activity (Beloozerova et al., 2010) not at all strenuous. Thus, it does not seem very likely that a difference in the proprioceptive afferentation between simple and ladder locomotion can be responsible for the entire volume and spectrum of discharge differences of MC, VL, and RE neurons during these two tasks. Nevertheless, in the majority of these neurons, discharge rate averages, peak values, depths of stride-related frequency modulation, and duration of PEFs are very different during ladder locomotion as compared to simple walking (Figure 9). We suggest that during ladder locomotion MC activity reflects processes that are involved in integration of visual information with ongoing locomotion and represents cortical commands that control stride length. These controls are different for different joints of the forelimb.

**Figure 11:**
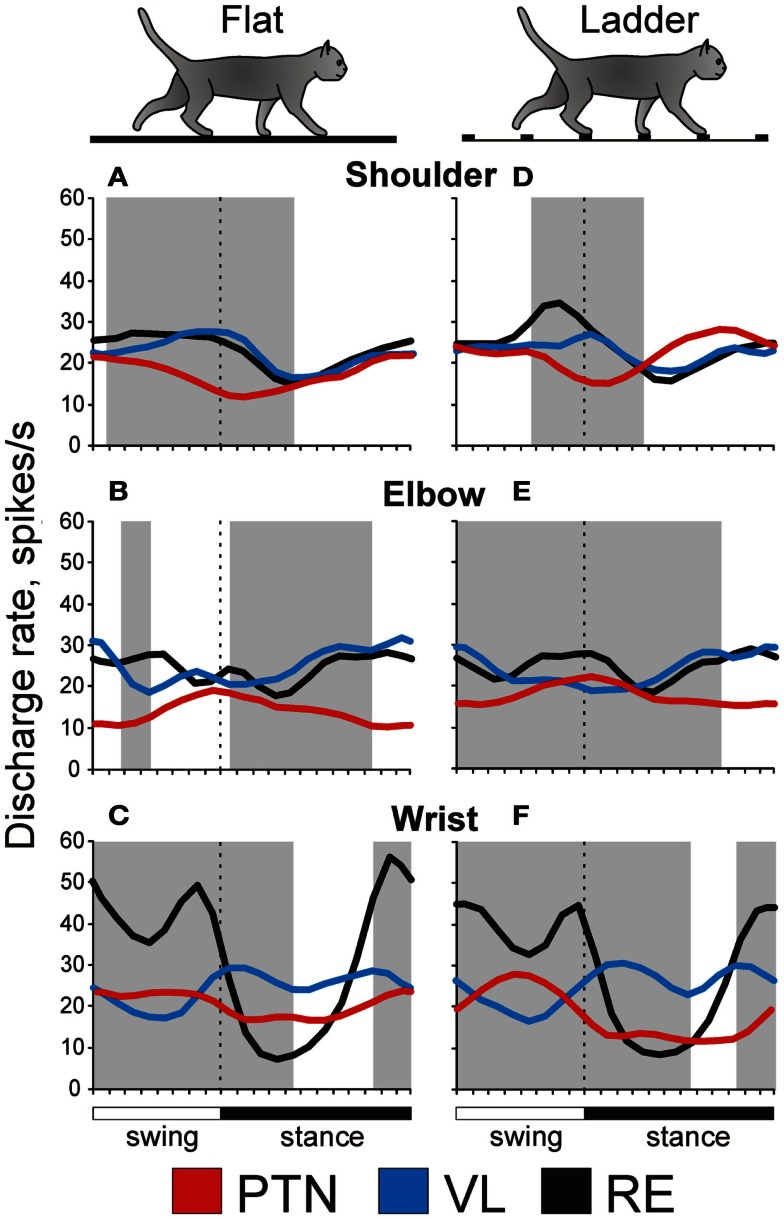
**Distinct thalamo-cortical controls for shoulder, elbow, and wrist during locomotion**. Red lines show population activities of shoulder-, elbow-, and wrist/paw-related neurons of the MC, blue lines show those of the VL, and black lines represent the corresponding activities of inhibitory neurons of the RE. Shaded are periods of the step cycle when the activities of the MC and VL are in anti-phase. (Data on the activity of PTNs, VL, and RE neurons are adapted with modifications from Stout and Beloozerova, 2012; Marlinski et al., 2012a,b, respectively).

Shoulder-related PTNs often increase their discharge rate and depth of modulation while reducing discharge duration. They typically do not change their preferred phase, but as a group become more active at the end of stance (Figures 10 and 11). Such activity modifications are consistent with the hypothesis that during precise stepping shoulder-related PTNs have a significant role in planning of limb transfer, which is believed to occur before the end of stance phase (Laurent and Thomson, 1988; Hollands and Marple-Horvat, 1996), as well as in the initial phases of limb transfer when adjustment of the foot trajectory is still possible (Reynolds and Day, 2005; Marigold et al., 2006). In addition, during the second half of stance, accurate paw placement of the opposing limb is taking place, and precise posture maintenance from the supporting limb is important to maintain balance. This could be another reason for shoulder-related PTNs, specifically those related to shoulder extension, to increase their activity and modulation during stance.

Wrist-related PTNs, whose activity was fairly evenly distributed throughout the cycle during simple locomotion, as a group became strongly modulated, exhibiting a prominent activity peak during swing (Figures 10 and 11). In contrast to shoulder-related PTNs, individual wrist-related PTNs often decreased discharge rate while also increasing depth of modulation and reducing their discharge duration. Such activity modifications are consistent with the hypothesis that wrist-related PTNs, specifically those related to the wrist plantar (ventral) flexion, are involved in distal limb transfer during accurate target stepping by ensuring greater plantar flexion of the wrist during the swing phase during ladder locomotion (Figure 2). It is well-known that activation of the MC results in contraction of more flexor than extensor muscles, and this rule holds during locomotion (Armstrong and Drew, 1985a).

Although both shoulder- and wrist-related PTNs often increase modulation during ladder locomotion as compared to simple walking, they generally do so using different mechanisms (Stout and Beloozerova, 2012). Shoulder-related PTNs often achieve an increase in modulation by increasing their peak discharge rate. This is likely to result in a more intensive signal to the spinal network, often along with a more specific timing of the discharge. Wrist-related PTNs achieve increases in the modulation chiefly by decreasing the firing outside of PEF, thus increasing the salience of the signal without making it more intense. This modification could specifically improve the temporal precision of the controls for limb transfer during a accurate stepping task.

In contrast to shoulder and wrist-related PTNs, upon transition from simple to ladder locomotion, elbow-related PTNs do not often increase the depth of modulation or discharge duration, but often increase discharge rate and change preferred phase. Their group activity becomes evenly distributed throughout the cycle during complex locomotion (Figures 10 and 11). The change in the preferred phase and the number of PEFs might reflect incorporation of visual information about the location of crosspieces into the CPG activity-based locomotor pattern, serving to “tweak” the limb into place to secure accurate stepping. The generally elevated activity of the elbow-related group is likely to enhance efficacy of their influence during complex locomotion task.

An effective way for PTNs to differentially influence different segments of the forelimb during locomotion is to influence individually the respective locomotion pattern formation networks in the spinal cord (McCrea and Rybak, 2008) by modulating the amplitude and potentially the timing of their output. Indeed, Asante and Martin (2010) recently found that in the mouse spinal projections from shoulder-, elbow-, and wrist-related areas in the MC primarily contact those spinal premotor circuits that connect to shoulder-, elbow-, and wrist-related motoneuron pools, respectively. Based on results of experiments with micro-stimulation in the MC, analogous mechanisms for control of limb segments have been previously suggested by Drew (1991) for the forelimb and by Bretzner and Drew (2005) for the hind limb of the cat.

### Signals from the VL-to-MC during accurate stepping contain integrated visuo-motor information for foot placement, differentiated by forelimb joint

How are motor cortical controls for shoulder, elbow, and wrist formed? The main subcortical input to the MC comes from the VL. The VL obtains locomotor CPG-generated information from the cerebellum, receives direct input from the spinal cord, and also receives visual information from the cerebellum and probably from the cortex. We found that during locomotion VL neurons discharge in a manner that is very suitable to contribute to the additional modulation of the activity in the MC that occurs during locomotion over complex terrain. Namely, the activity of VL neurons with one PEF is modulated more strongly on the ladder than during simple locomotion, the overwhelming majority of individual VL neurons change their discharges upon transition from simple to ladder locomotion, and the dominant change, similar to that in the MC, is an increase in the depth and temporal precision of the modulation.

What is the content of information conveyed by the VL to the motor cortex during ladder locomotion? Considering the rather similar limb motor patterns (Beloozerova et al., 2010) but dramatically different gaze behaviors (Rivers et al., 2009, 2010) in the two locomotion tasks, we suggest that at least a part of the differences in discharges of VL neurons during simple and ladder locomotion reflects differences in processing of visual information during these two tasks, as well as the changes in motor commands made on the basis of visual information. During locomotion in complex environments, visual information about the position of the stepping target is first processed through visual networks and then at some point is incorporated into the basic locomotion rhythm in order to guide the limb. From this point on it becomes integrated “visuo-motor” information that, in the afferent sense, is “(processed) visual information,” while in the efferent sense it is a “limb control signal” reflecting preparation of the movement. It has been suggested that visual information about the environment is integrated with movement-related information in the cerebellum, and then funneled to the motor cortex via the VL for control of limb movements (Glickstein and Gibson, 1976; Stein and Glickstein, 1992; Glickstein, 2000). Our data indicate, however, that the VL is more than a simple relay for signals passing to the MC during ladder locomotion. Many of VL neurons discharge in different phases of the cycle during simple and ladder locomotion. This shows that information related to the complex environment changes the basic locomotion-related discharge pattern of VL neurons. In our original research report we have described five major modes of this integration (Marlinski et al., 2012a).

### The RE differentially gates TC signals depending on locomotion task

Two thirds of RE neurons change at least one aspect of their activity upon transition from simple to ladder locomotion. This indicates that participation of the RE in shaping of VL signals going to the MC depends on the task. The mean and peak activities in 33–37% of RE neurons during ladder locomotion are different from those during simple walking. This signifies differences in the intensity of regulation of the VL-to-MC transmission between two tasks. Differences in the depth of modulation in 40% of RE neurons mean differences in the salience of the RE to VL influence. Differences in the preferred phase, duration of PEFs and/or in the number of PEFs mean differences to the timing of RE influences on the thalamo-cortical signal transmission, and these are often seen in RE neurons between two tasks.

## Conclusion

In this review, we have presented the results of a series of studies that examined the differences in the activities of shoulder-, elbow-, and wrist/paw-related neurons in the thalamo-cortical network for locomotion. Substantial differences were found both between the subpopulations of neurons with different receptive fields within each of the MC, VL, and RE, as well as between neurons with similar receptive fields residing in different motor centers. We conclude that the thalamo-cortical network for locomotion processes information related to different segments of the forelimb differently and exerts distinct controls over shoulder, elbow, and wrist. We hypothesize that this contributes to an effective control of a global limb parameter, the length of the stride, which results in a great reduction in variability of paw placement during accurate stepping. It is one of manifestations of a modular organization of control for locomotion. The efficacy and contribution of synaptic connections between neurons with similar and dissimilar receptive fields in different sites in the thalamus and cortex need to be determined, however, to further reveal the operation of thalamo-cortical neuronal network during locomotion.

## Conflict of Interest Statement

The authors declare that the research was conducted in the absence of any commercial or financial relationships that could be construed as a potential conflict of interest.
